# Rapid LC-MS Based High-Throughput Screening Method, Affording No False Positives or False Negatives, Identifies a New Inhibitor for Carbonic Anhydrase

**DOI:** 10.1038/s41598-017-08602-w

**Published:** 2017-09-04

**Authors:** Kasun P. Imaduwage, Jude Lakbub, Eden P. Go, Heather Desaire

**Affiliations:** The Ralph N. Adams Institute for Bioanalytical Chemistry and Department of Chemistry, University of Kansas, 2030 Becker Drive, Lawrence, KS 66047 USA

## Abstract

Developing effective high-throughput screening (HTS) methods is of paramount importance in the early stage of drug discovery. While rugged and robust assays may be easily developed for certain enzymes, HTS assays designed to identify ligands that block protein binding are much more challenging to develop; attenuating the number of false positives and false negatives under high-throughput screening conditions is particularly difficult. We describe an MS-based HTS workflow that addresses these challenges. The assay mitigates false positives by selectively identifying positive hits exclusively when a ligand at the binding site of interest is displaced; it mitigates false negatives by detecting a reporter compound that ionizes well, not by detecting the ligand binder, which may not ionize. The method was validated by detecting known binders of three proteins, pepsin, maltose binding protein (MBP), and carbonic anhydrase (CA) in the presence of hundreds of non-binders. We also identified a novel CA binder, pifithrin-µ, which could not have been identified by any other MS-based assay because of its poor ionization efficiency. This new method addresses many of the challenges that are currently encountered during high-throughput screening.

## Introduction

Identifying potent and high affinity ligands for target proteins is a vital first step in drug development. Generally, these molecules are identified during high-throughput screening campaigns, and the most commonly used analysis methods are florescence^1, 2^, chemiluminescence^3^, and surface plasmon resonance (SPR)^4^. All of these methods suffer from the same limitation: modification of the analytes or the protein is typically necessary for detection. Thus, assay development can be laborious, and the molecular labels themselves can alter the integrity of the binding between ligands and the target protein, leading to false positives and false negatives. Although nuclear magnetic resonance (NMR) is an alternative label free spectroscopy technique^5, 6^, it requires substantially more protein, and data analysis is laborious, resulting in very low throughput. As a result of the continued need for fast, label-free detection methods, mass spectrometry (MS) based screening techniques are becoming more common^7^. The key advantages of an MS-based method are the label free nature, high sensitivity, and the ability to differentiate ligands based on the analytes’ masses.

Various MS-based techniques have been developed over the past few decades, and these methods can be subdivided into those approaches that either do or do not require a chromatographic separation step as part of the analysis. The most commonly used MS-based methods without chromatography rely on MALDI-TOF-MS^8–10^. This MS platform is fast and sensitive, but matrix interferences and poor reproducibility are some shortcomings that can contribute to false positive and false negative identifications^11, 12^. Common MS-based HTS methods that incorporate a separation step allow for more compounds to be interrogated at one time; example separation platforms include size-exclusion chromatography coupled with reverse phase chromatography-MS (SEC-RPC-MS)^13^, ultrafiltration-MS^14^, gel filtration-MS^15^, frontal affinity chromatography-MS^16, 17^, and affinity capillary electrophoresis-MS^18^. In these methods, binders are generally identified either by direct detection of the protein-ligand complex^13^, or by detection of bound compounds after dissociation of the protein-ligand complex^15–17, 19^, or by detection of unbound compounds compared with a control^19^. The most common limitations to these approaches are false positives, due to non-specific binding, false negatives, due to the presence of non-ionizable compounds, the need for large amounts of target protein, and insufficient throughput^13, 15, 20, 21^.

The most commonly used MS-based HTS method is the affinity selection–MS screening method (ASMS). This approach currently has the best balance of strengths and limitations, and it can be used for screening over 1 × 10^5^ compounds per day^22–25^. While this level of throughput is a clear advantage, false positives, which are due to nonspecific binding of small molecules, introduce a significant disadvantage^13, 15, 20^. As such, the hits from this type of screen need to be validated with an alternative screening technique in order to completely rule out possible false positives. Additionally, the method is not able to detect hits that do not ionize, so false negatives are also a concern.

Recently, we reported a novel MS-based HTS method, High-Affinity Mass Spectrometry screening (HAMS), which uniquely evades the detection of false positive hits^26^. However, the method requires acquisition of two LC-MS datasets per set of 350 compounds, and this requirement limits throughput. Therefore, further development in MS-based high-throughput screening is needed to overcome the field’s current limitations.

Herein, a new approach is developed that fills the existing technology gaps in high-throughput screening. This method avoids false positives and false negatives; it can be used to screen over 10,000 compounds per day, while consuming limited protein quantities. Target proteins are incubated with a known ionizable weak binder (reporter molecule), and the complex is then introduced to a batch of library compounds, while an equimolar amount of the complex, without the library compounds, is used as a control sample. LC-MS is used to detect the reporter molecule. If a stronger binder is present in the library, the signal of the reporter molecule increases compared to the molecule’s signal in the control samples. In this way, a binding event is measurable, even if the strong-binding ligand is not detectable by mass spectrometry; in other MS-based assays, non-ionizing compounds result in false negatives. In addition, the assay has very modest protein requirements, consuming ng of protein per compound analyzed. Finally, the analysis time for the method described herein is a quick 10 minutes per batch of 300–400 compounds, which extrapolates to well over 10,000 compounds per day. Overall, this MS-based HTS method meets the current needs of the drug discovery field better than any existing MS-based method. Furthermore, the assay’s value is demonstrated by identifying a new inhibitor for carbonic anhydrase, a therapeutically valuable protein.

## Methods

### Reagents

FDA approved drug compounds (1280 compounds) were purchased from Sigma Aldrich (St. Louis, MO). The library was provided as a single compound per vial (LOPAC1280-small scale), dissolved in DMSO at a concentration of 10 mM. Pepsin, Aminolink Plus coupling resin, and disposable plastic columns were obtained from Pierce Biotechnology, Inc. (Rockford, IL). Pierce^TM^ NHS-Activated Magnetic Beads were purchased from Thermo Scientific (Workford, IL). Maltose binding protein and carbonic anhydrase were purchased from My BioSource (San Diego, CA) and Sigma Aldrich (Milwaukee, WI), respectively. Nitrocellulose drop dialysis membranes were purchased from Fisher Scientific (Houston, TX).

### Protein immobilization onto Aminolink Plus coupling resin

Immobilizations of carbonic anhydrase (CA) and pepsin were carried out by adjusting a previously published procedure^26^. Each protein was maintained at its optimal pH. Therefore, for CA the coupling buffer was phosphate buffered saline (PBS) (0.1 M, pH 7.4), the blocking buffer was Tris HCl (1.0 M, pH 7.4), the incubation buffer was ammonium acetate (0.02 M, pH 7.4), and the wash buffer was 1 M NaCl in coupling buffer. Similarly, for pepsin, citric acid and NaHPO_4_ (0.1 M, pH 4.5) were used as the coupling buffer; the blocking buffer was Tris HCl (1.0 M, pH 4.5), the incubation buffer was ammonium acetate (0.02 M, pH 4.0), and the wash buffer was 1 M NaCl in coupling buffer. All of the pepsin assays were carried out through immobilization of 1 or 2 µg of pepsin with 25 µL of resin beads. Similarly, 10 µg of carbonic anhydrase was coupled to 25 µL of resin beads.

Both CA and pepsin were dissolved in 100 µL of coupling buffer before drop dialysis was conducted. Dialysis of the dissolved proteins with the coupling buffer was performed over 1 h using a 0.025 mm nitrocellulose drop dialysis membrane. Subsequently, the protein was added to a disposable plastic column that was filled with coupling resin that had been washed with 2 mL of coupling buffer. Then, a solution of 1 M NaCNBH_4,_ in coupling buffer, was added to the resin solution until a final concentration of 50 mM NaCNBH_4_ was reached. The column and resin solution was rocked overnight, then washed with 10 mL of coupling buffer, followed by 5 mL of blocking buffer. Next, 1 mL of blocking buffer was added, and 1 M NaCNBH_4_ was added again until a final concentration of 50 mM NaCNBH_4_ was reached. The mixture was rocked for 2 h, and the column was washed with 10 mL of coupling buffer, 10 mL of wash buffer, and 15 mL of incubation buffer, in that order. Finally, the immobilized protein was transferred to Eppendorf tubes for immediate incubation with the library compounds.

### Preparation of library compounds for binding experiment

To prepare the 100-compound libraries, 2 µL of each compound’s stock solution (10 mM) was combined into 100 compound batches at a final concentration of 100 µM. Then, 2 µL of the 100 µM batches (100 compounds each) was diluted with incubation buffer, specific to each protein, to obtain a 25 µM stock solution. When preparing the library of 400 compounds, 2 µL solutions from each of the four libraries of 100 compounds were combined, achieving a final concentration of 25 µM. Then, for both libraries of 100 or 400 compounds, 2 µL of the 25 µM library was diluted to 337.5 nM using the incubation buffer, specific to each protein.

### Binding experiment using proteins immobilized on Aminolink coupling resin

Immobilized pepsin and CA were incubated with 200 μL of 300 nM known weak binders, pepsinothipsongen or methoxzolamide respectively. After incubation, tubes were centrifuged at 3000 × g for 5 minutes; the supernatant was removed, and the immobilized proteins were washed twice with 500 μL of incubation buffer. Next, 200 µL of the 100 or 400 compound library mixture was added to 25 µL of immobilized protein mixture, bringing the final concentration of the library compounds down to 300 nM. The mixture was rocked for 1 h at room temperature, then centrifuged at 3000 × g for 5 minutes; the supernatant was removed and used directly for LC-MS analysis.

### Binding experiment for low-affinity binders

Maltose binding protein (MBP) was immobilized on N-hydroxysuccinimide (NHS)-activated magnetic beads by following the manufacturer’s protocol. Briefly, drop dialysis was conducted on MBP in PBS buffer (0.1 M, pH 7.4). Then, 100 µL of magnetic beads were washed according to the manufacturer’s instructions and 20 µg of MBP in coupling buffer (PBS, 0.1 M, pH 7.4) was added. The mixture was slowly rocked overnight at room temperature. Subsequently, the supernatant was removed, and the immobilized proteins were washed with 1 mL of coupling buffer. Then, 300 µL of quenching buffer (Tris HCl, 1.0 M, pH 7.4) was added and the mixture was rocked for 2 hours. The supernatant was removed, and immobilized proteins were washed with 300 µL of washing buffer (1 M NaCl in coupling buffer) followed by 2 mL of ammonium acetate (0.02 M, pH 8.0) incubation buffer. The supernatant was removed again and 50 µL of 50 nM maltose, a known weak binder, was added. The mixture was rocked for an additional two hours at room temperature. After removing the supernatant, 50 µL of the 400 compound library (300 nM) was added, and the mixture was rocked again for one hour. After incubation, the supernatant was removed and used directly in the LC-MS analysis.

### Positive and Negative Controls

Positive and negative control samples were prepared, as explained in the *binding experiments* section above, for high and low affinity ligands, except libraries with a known strong binder were used as positive control samples. For each positive control, 401 library compounds were used. Negative control samples contained either buffer only or 400 non-binding compounds. Supernatants for both controls were removed and subjected to LC-MS analysis.

### LC/MS Analysis

An Acquity UPLC system (Waters Corporation, Milford, MA) coupled to an Orbitrap Velos Pro Mass Spectrometer was used for liquid chromatography/mass spectrometry analysis. Mobile phase A was 99.9% water with 0.1% formic acid, and Mobile Phase B was 99.9% MeOH with 0.1% formic acid. Five microliters of the supernatant was injected onto a C_18_ Hypersil Gold column (Particle Size: 5 µm; 1 mm *i.d* × 100 mm, 175 Å, Thermo Electron Corporation, Thermo Fisher Scientific, Pittsburgh, PA) at a flow rate of 50 μL/min. The multi-step gradient used for assays, excluding any analyses for MBP, was as follows: 100% solvent A for 1 min, then a linear increase of B to 80% in 4 min, followed by the linear increase of B to 95% in the next 3 min; then B was maintained at 95% for an additional 30 seconds, finally a linear decrease of B to 0% in the next 30 seconds, where the solvent composition was maintained for another 2 min. The following multi-step gradient was used for MBP assays: 100% solvent A for 3 min, then a linear increase of B to 80% in 2 min, followed by the linear increase of B to 95% in the next 3 min, then B was maintained at 95% for an additional 30 seconds, finally, a linear decrease of B to 0% in next 30 seconds, where the column was maintained for another 2 min. The eluent was diverted to waste for 7 minutes at the beginning of each run, except for the experiments conducted with MBP, where the divert time was reduced to 1 min. The mass spectrometer was operated in the positive ion mode with a 3 kV potential on the ESI needle, and the capillary temperature was set at 250 °C. Full scan MS data were obtained at a mass range of *m/z* 200–700 using the Orbitrap mass analyzer at a resolution of 30,000 for *m/z* 400.

All peak areas were calculated using the extracted ion chromatograms from the.raw data files for each compound. The extracted ion chromatograms were generated for the monoisotopic peaks of protonated or/and sodiated adducts for every reporter molecule as follows: pepsinostreptin (*m/z* 672.4548), methoxzolamide (*m/z* 237.0110), maltose (*m/z* 343.1235, 365.1054), and chlorothiazide (*m/z* 295.9561). The peak area for each extracted ion chromatogram was calculated using the software (Xcaliber) supported algorithm, Genesis.

### Calculation of IC50 of pifithrin-µ

IC50 was determined by plotting the % inhibition versus the log of the concentration of the inhibitor, adapting a well-established method^27^. Experiments were repeated in triplicate. For each trial, 100 µL of 0.125 mg/mL carbonic anhydrase (4.3 µM) in Tris buffer (pH 8.4) was used. First, the protein solution was mixed with 9.9 mL Tris buffer containing different concentrations of inhibitor. The inhibitor concentrations were 0 nM, 1 nM, 5 nM, 10 nM, 20 nM, 40 nM, 60 nM, 100 nM, 130 nM, 200 nM, 350 nM, 500 nM, 1 µM, 2 µM, 4 µM, 6 µM, 8 µM, and 10 µM. These protein-inhibitor mixtures were incubated for 1 hour on a rocking platform at 4 °C (on ice). After incubation, 4 mL of carbonated Tris buffer, which was pre-equilibrated at 4 °C (on ice), was added with stirring. The final concentration of the protein in the reaction mixture was 0.03 µM. Simultaneously, the pH of the solution was recorded over time. The time required for the pH of the solution to change from 8.4 to 6.4 was recorded in order to calculate the % inhibition. The entire reaction was carried out at 4 °C (on ice) with stirring. The % inhibition was calculated using the following equation: % inhibition = [(normal activity − inhibited activity)]/(normal activity)].

### Data Availability

The raw data files analyzed during the current study are available from the corresponding author upon reasonable request.

## Result and Discussion

### Method Overview

**Figure 1:**
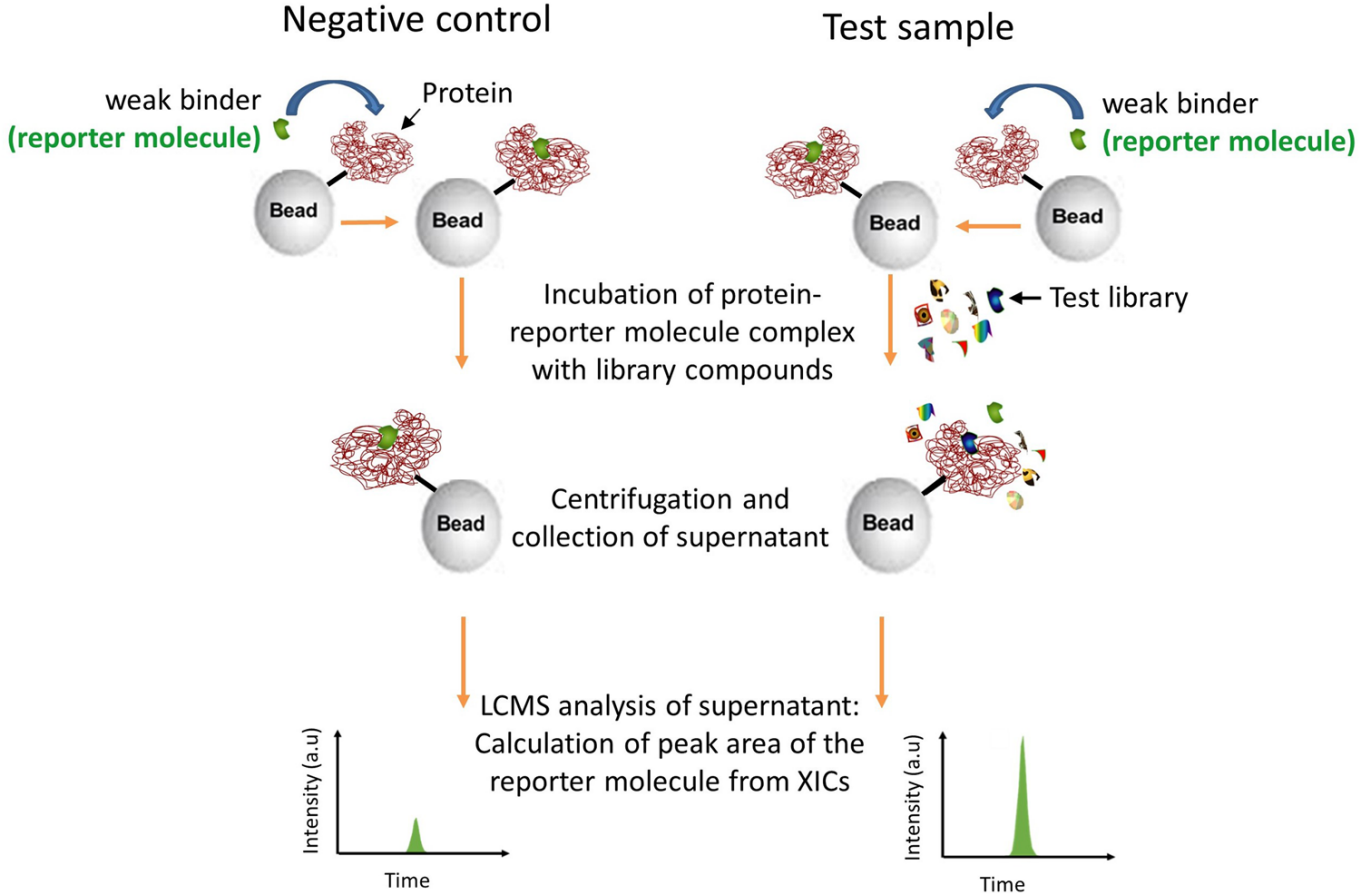
Experimental workflow. The weaker binder (reporter molecule) is incubated with the immobilized protein, and the excess reporter molecule was washed off. The library compounds are added to the test sample. During validation experiments, a known strong binder is spiked into the library, generating a positive control sample. When present, a strong binder replaces the reporter molecule on the binding site of the protein, and the concentration of the reporter molecule increases in the supernatant relative to the negative control. Supernatants for both the negative control and the test samples are analyzed for the presence of reporter molecule, and the peak area of the reporter molecule is used to calculate the Z’ factor during validation experiments.

Figure 1 describes the workflow for the assay developed herein. Immobilized protein of interest is first incubated with a compound known to bind to the target protein; hereafter, this compound is referred to as “the weaker binder” or “reporter molecule”, although its binding affinity can be in the micromolar to picomolar range. After incubation, excess weaker binder is removed with the incubation buffer, and the immobilized protein/weaker binder complex is incubated with the library compounds of interest; typically batches of 400 compounds are tested. If the library contains a stronger binder, the high affinity binder competes for the same binding site in the protein with the known weaker binder. The weaker binder is, therefore, displaced from the complex. Hence, the impact of a stronger binding compound being present is that the concentration of the weaker binder in the supernatant increases compared to that of the negative control, when no library compounds are added. Although no strong binders are present in the negative control, a certain degree of dissociation of the weaker binder from the protein occurs, depending on the concentrations of the protein and ligand and the strength of the binding interaction. It should be noted that the purpose of this assay is to identify ligands with optimized binding affinity for protein targets that have known, but sub-optimal, binders. We note that the only additional property necessary for the weaker binder (reporter molecule) is that it is detectable by mass spectrometry. It does not, for example, need to be fluorescent or detectable by any other means. For many druggable targets, binding compounds are known, and we provide three relevant examples herein. For a protein with no known binding compounds, a complementary assay could be used to identify a weak-binding reporter molecule^26^.

To demonstrate that this workflow provides sufficient discriminatory power between ligand sets that do or do not have a strong binder present, several control experiments were completed on different protein/ligand systems. During these control experiments, a stronger binder is always included in the positive control. Three model proteins (pepsin, carbonic anhydrase (CA), and maltose binding protein (MBP)), their known weaker binders, and their known strong binders were used in three separate proof-of-concept experiments. In each case, the peak area of the weaker binder in the positive control was compared to the signal for the weaker binder in the negative control. These peak areas were used to calculate a Z’ factor, which is a statistical measure used to determine the quality of an HTS assay^28^. The Z’ factor is equal to: 1−[3(σ_p_ +  σ_n_)/(|μ_p_ − μ_n_|)]; where σ is the standard deviation of the measurement, and μ is the mean for the measurement. Signals for both positive controls (p) and negative controls (n) are required to calculate the Z’ factor. If the Z’ factor is between 0.5 and 1.0, the assay is considered excellent, whereas if the calculated Z’ factor is <0.5 and >0, the method is not considered to be very effective, by most standards^28^. Each experiment is discussed in detail next, starting with the proteins that have known tight-binding inhibitors.

### Identification of high affinity binders (pM to nM range)

The assay was first validated using proteins that have known high-affinity ligands. Immobilized pepsin and CA were incubated with their known binding ligands (reporter molecules), pepsinostreptin and methoxzolamide, respectively. For negative controls, the protein/ligand complexes were each incubated with a library of 400 compounds that did not contain any strong binders, while the validation samples (positive controls) were prepared by incubating the protein-reporter molecule complexes with the same library as the negative control and also the protein’s known stronger binder. One strong binder (pepstatin A, K_d_ = 50 pM) was used for pepsin, while two strong binders (Ethoxzolamide, K_d_ = 750 pM, and Brinzolamide K_d_ = 3 nM.), were used in two separate experiments for CA. After incubation with the library compounds, the negative controls and the positive controls were each centrifuged, and the supernatants were analyzed using LC-MS. In each case, detection and quantification of the reporter molecule, the weaker binder, was achieved in a 10 minute chromatographic run. Five replicates were completed for each protein.

**Figure 2:**
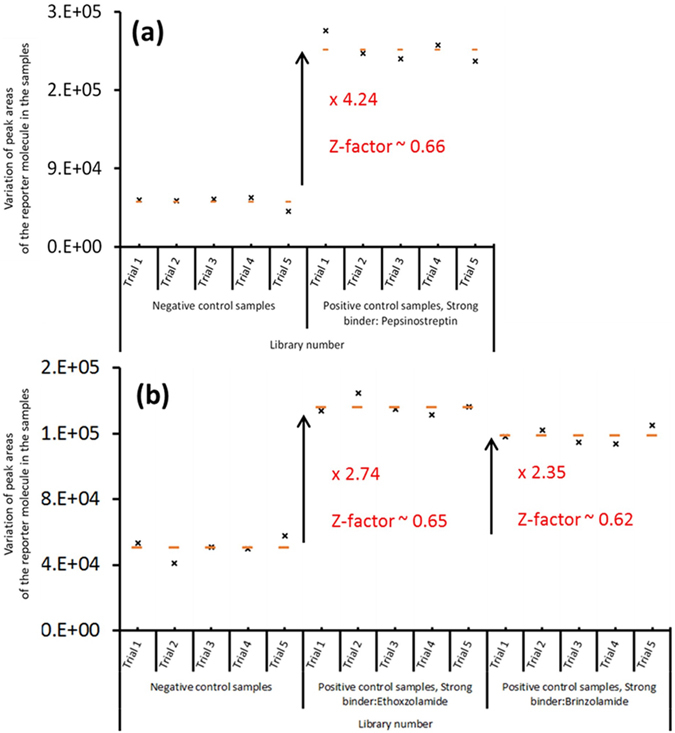
Results of the validation experiments for (**a**) pepsin and (**b**) carbonic anhydrase. The calculated Z’ factors indicate that the assays have appropriate discriminatory power for a high-throughput screen.

Figure 2 shows the results of these experiments. For both proteins, a significant increase of the reporter molecules was detected in the supernatant of the positive controls for all five trials compared to the five negative controls. This result indicates that the strong binders in the positive controls displaced the weaker binders in the active sites of the proteins, leading to an increase of the weaker binders in the supernatant of the positive controls. The peak area of pepsin’s weaker binder is approximately four times larger in the positive controls than the negative controls (Fig. 2a). The peak areas for the weaker binder in both the positive and negative controls are reproducible; and the calculated Z’ factor is >0.6, indicating that the assay is appropriate for high throughput screening of strong binders in the pM range.

Figure 2b shows similar results for the two different CA binding experiments. The Z’ factors for both experiments are also greater than 0.6, falling into the “excellent” category for HTS assays of strong binders in the nM range. In addition, Fig. 2b also shows slight differences in the peak areas of the reporter molecule for the two different strong binders for CA. The variation in peak areas in these two experiments is expected due to the difference between the dissociation constants of the two binders. In other words, the larger the peak area for the reporter molecule indicates a compound with stronger binding affinity is present. In Fig. 2b, the binding affinity for ethoxazolamide is stronger than that of brinzolamide. These trials required only 1 µg (~30 pmol) of pepsin and 10 µg (~310 pmol) of CA to screen 400 compounds in each library. While the CA assay, which profiled a lower affinity ligand, required more protein, this protein quantity is still significantly lower than the commonly used amount for the screening batches of 400 compounds against CA^25^.

We note here that this assay does not indicate *which* of the compounds in the positive control was the binding agent responsible for displacing the weak binder. Rather, it simply indicates that a strong binder is present in the positive control. In the case of the data acquired in Fig. 2a, for example, the assay indicates that pepsinostreptin is the strong binder because all the other compounds were present in the negative control.

During screening, where 400 different compounds could be responsible for displacing the reporter molecule, a well that produces a positive hit must be further interrogated to identify which of the compounds is the strong binder. The binder can be identified in a variety of ways. For example, we recommend using a complementary assay that we recently described, coined “HAMS” (high-affinity mass spectrometry screening)^26^. In that assay, the tightest binder in sets of 400 compounds is identified using a screening procedure that is similar to the workflow described here. A key difference in the HAMS assay is that instead of detecting an *increase* in reporter ion concentration, the MS signal for all the compounds in the well are monitored simultaneously, and the binder is the compound whose signal *decreases completely*, compared to a control. This assay can be performed at a rate of about an hour per well, so it does not have the throughput of the assay described herein, but is likely best used as a complementary assay that identifies which compound was the binder. In a hypothetical experiment where 40,000 compounds are screened, (400 at a time) the assay described herein could be performed in ~16 hours, or 1,000 minutes (100 wells *10 minutes). If five of the wells contain a positive hit, the five lead compounds in those wells could be determined with a re-screening that would comprise an additional hour per well, or five additional hours. Thus, five strong binders, out of 40,000, could be identified in <24 hours. In an actual screen, discussed below, we were able to identify a novel inhibitor without a complementary re-screening approach. The approach we describe at the end of this manuscript is a second viable approach for identifying hits generated by the screening method described here.

### Identification of transient binders (µM range)

After successfully applying the assay to screen for tight-binding ligands, we tested whether the same procedure could be used to identify lower-affinity ligands. In these experiments, maltose binding protein (MBP) was the target; maltose (K_d_ of ~3 µM) was used as the weaker binder (reporter molecule), while maltotriose (K_d_ = 0.2 µM) was the stronger binder. As before, the stronger binder was spiked into the positive controls, but not the negative controls. No significant difference in the signal of the reporter ion in the positive and negative controls was detectable in these experiments. In considering the potential reasons for the experiment’s failure, we suspected that the centrifugation step, which is necessary to separate the immobilized protein from the supernatant, was causing the weakly bound reporter molecule to dissociate from the complex to a significant degree, even when a stronger binder was not present.

To address this problem, magnetic beads were used to immobilize the protein, instead of agarose beads. Since magnetic beads were used, no centrifugation was done, thereby minimizing the dissociation of reporter molecules from immobilized protein due to the centrifugal force.

**Figure 3:**
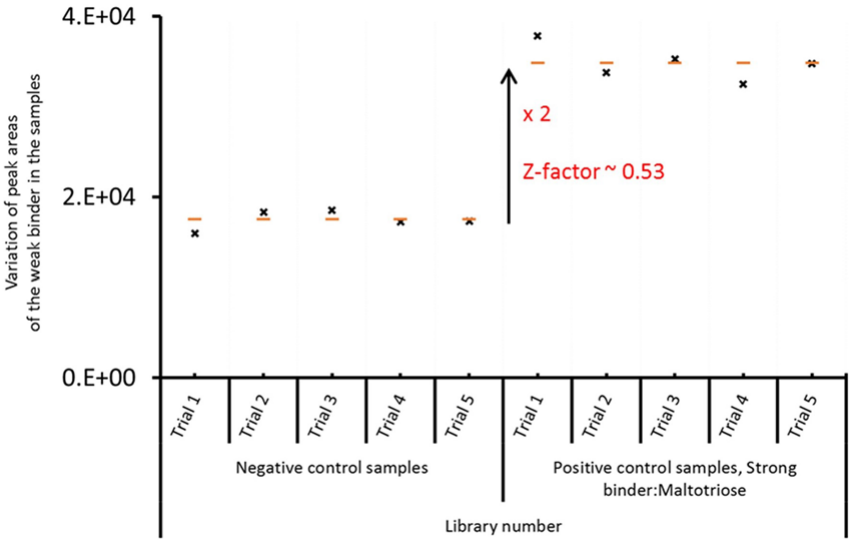
Results of the validation experiment for maltose binding protein. The calculated Z’ factor indicates that the assay has appropriate discriminatory power for a high-throughput screen.

After making the aforementioned changes, MBP was immobilized on magnetic beads followed by incubation (loading) with the reporter molecule, maltose. Subsequently, the supernatant was removed without centrifugation and the MBP-maltose complex was incubated with library compounds. A library of 400 compounds was used for the negative control; for the positive control, maltoriose, the stronger binder, was spiked into the same library. Finally, the protein was sequestered from the supernatant without centrifugation, and the supernatant was then analyzed for the reporter molecule using LC-MS. Five trials were conducted for each control. Figure 3 shows the results of the experiments. The increase in the amount of reporter molecules in the supernatant of the positive controls compared to negative controls indicates the experiment was a success. The calculated Z’ factor for the experiments was 0.53, showing that the assay can be applied for high-throughput screening of low-affinity binders.

### Addressing false negatives

**Figure 4:**
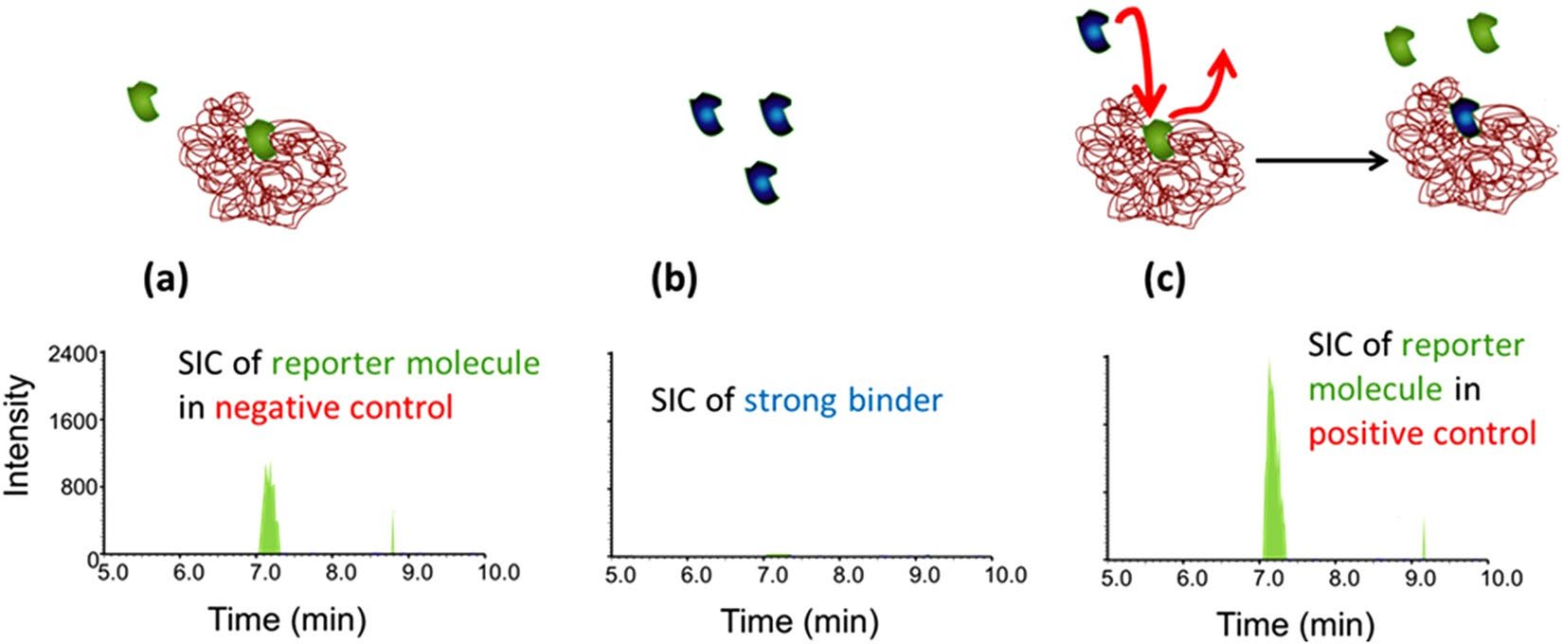
(**a**) Selected ion chromatogram (SIC) of the weak-binding reporter molecule, chlorothiazide, in the supernatant of the negative control. (**b**) SIC of the non-ionizing strong binder, acetazolamide. (**c**) SIC of the reporter molecule in the supernatant after the strong binder, acetazolamide, had been added. This experiment demonstrates that the assay can detect binders that do not ionize. In other MS-based assays, these compounds are either un-assayable or they are false negatives.

A significant limitation of current MS-based HTS methods is the high rate of false negative identifications due to non-ionizable compounds not being detected in MS-based assays. We carried out an experiment to demonstrate that the method described herein overcomes this limitation. The experiment was conducted using carbonic anhydrase (CA) as the model protein, chlorothiazide as the reporter molecule, and acetazolamide, which is a non-ionizable molecule, as the strong binder. Figure 4 shows a pictoral description of the experiment along with the resulting data. Figure 4a shows the signal for the reporter molecule, chlorothiazide, when no strong binders are present in the assay; this is the negative control. The strong binder, acetazolimide, cannot be detected by mass spectrometry, as shown in Figure  4b; however, when acetazolamide is incubated with a CA-chlorothiazide complex, a significant increase of the peak area of the reporter molecule, chlorothiazide, is detectable. This increase in signal can be seen by comparing the data in Fig. 4a, the negative control, to the data in Fig. 4c, when acetazolimide is present. The increase in concentration of the reporter molecule, chlorothiazide, in the supernatant is due to the competitive binding of the non-ionizable strong binder, acetazolamide, to carbonic anhydrase. Hence, the assay can identify the presence of strong binders from a pool of library compounds, irrespective of whether the compounds ionize well or not.

### Screening for new inhibitors

**Figure 5:**
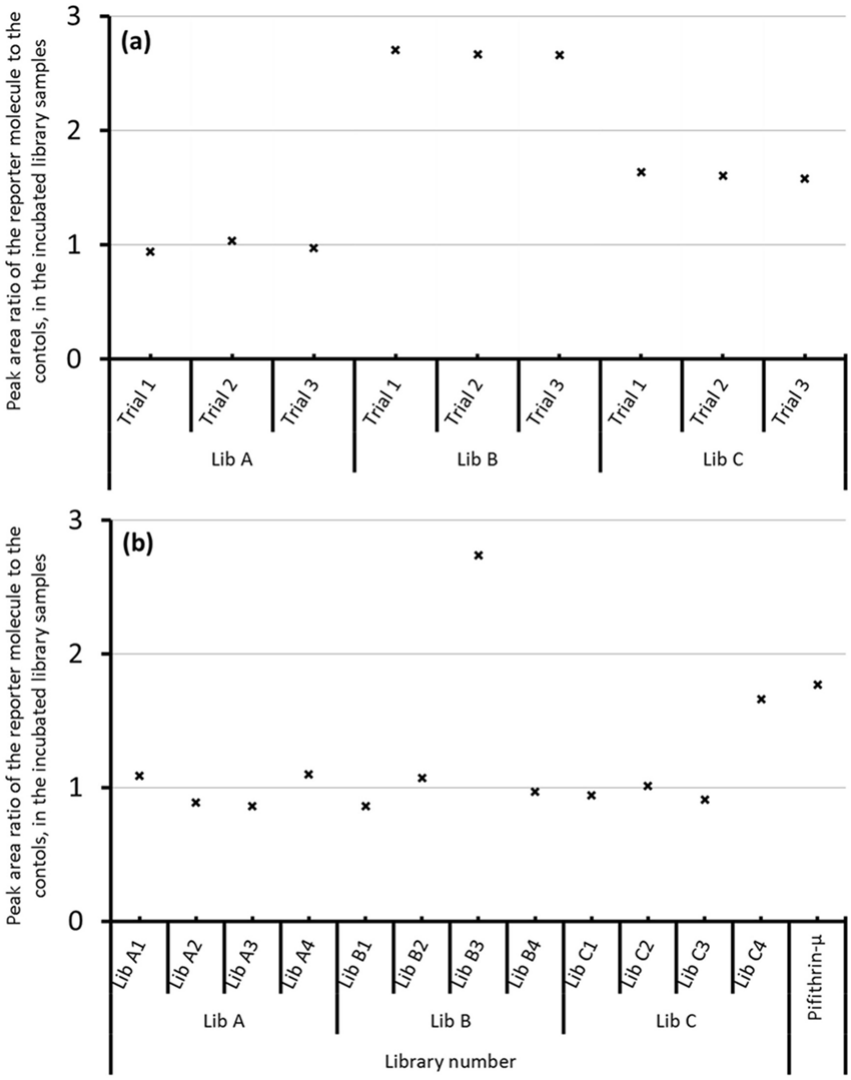
Screening assay for CA binders with methoxzolamide as the reporter molecule. (**a**) Tests conducted with 400 compound libraries. (**b**) Tests with 100 compound libraries and with pifithrin-µ. A strong binder, ethoxzolamide, was spiked into Library B (400 compound set) and Library 3B (100 compound set) to verify that a positive response would be readily detectable when a strong binder was present. The positive response for Library C and Library C4 resulted from pifithrin-µ, a CA inhibitor that was newly-identified in this assay.

Because the new assay had demonstrated strong performance metrics during method development, we next tested a well-known target protein against a moderate sized library of 1200 compounds, in order to both verify that a known inhibitor could be readily identified and also to pan for unknown inhibitors. A known CA binder, ethoxzolamide, was spiked into one set of four hundred compounds, and two other compound sets, each containing 400 compounds, were also prepared. These three batches were used to screen for CA binders. The compound set with the known inhibitor, henceforth referred to as Library B, was effective at out-competing the weak inhibitor that had initially been pre-loaded into the CA binding site. The weaker binder was readily detectable in the assay supernatant, signaling that a compound in the library was out-competing it for CA binding. Figure 5a shows that in three replicate trials, the concentration of the weaker binder was almost three times that of the concentration of the control sample, where no library compounds competed for binding. While the data in Fig. 5 do not say *which* compound in Library B was the strong binder, we have previously demonstrated that this inhibitor (ethoxzolamide) can easily be identified as the strong binder in a batch of ~400 compounds, using the recently published HAMS screening method, where the highest-affinity ligand (of the 400 in the well) is identified in a complementary screen^26^.

Figure 5a also shows results for the two other sets of four hundred compounds, which are referred to as Library A and Library C. Neither one of these sets had a known CA inhibitor present. With no inhibitor present, each of the data points in the graph in Fig. 5a should have Y coordinates of approximately one. These data points represent a ratio of the reporter ion’s signal in the presence and absence of the library compounds. When no strong binder is present, the reporter ion’s signal should be the same, regardless of whether or not other compounds are present; therefore, the ratio comparing when compounds are present to when they are absent, should be one if no inhibitor is present. For Library A, all three replicates gave such a value, near 1.0. However, the signal for the reporter ion was much higher when Library C was added. These results indicate that some compound in Library C is out-competing the reporter molecule for the CA binding site and is therefore inhibiting CA binding. This exciting result suggested that a new CA inhibitor was identified by screening just 1200 well-known compounds against a well-studied target protein.

To further validate the results of the initial screen, and to gain more insight into the new inhibitor that had been identified, Library A, B, and C (Fig. 5a) were divided into four batches with 100 compounds each (Fig. 5b), and the same experiment was conducted as described above. The known strong binder was spiked in to Library B3 (Fig. 5b). Ten of the 100 compound libraries indicated that no strong binding compounds were present; this finding emphasizes the assays power in evading false positives. The only two libraries that contained hits were Library B3, which had been spiked with the known strong binder, ethoxzolamide, and Library C4 (Fig. 5b). The high response from Library C4 was consistent with our previous experiment, and it further indicated that a compound in Library C4 was binding to CA and displacing the weaker-binding ligand.

After we had identified the set of 100 compounds that likely contained a new hit for CA, we attempted to identify the inhibitor based on structural similarity to known CA inhibitors. If the inhibitor could be tentatively identified based on structural similarity to known inhibitors, then an alternative screening procedure would not need to be implemented. The chemical structures of the 100 compounds in Library C4 were compared with well-known CA inhibitors. The compound pifithrin-µ was found to have considerable functional group similarities with other CA inhibitors (see Supporting Fig. 1). However, to our knowledge, pifithrin-µ has not been reported as a CA inhibitor. To confirm that pifithrin-µ is responsible for the competitive binding effect, another screen was carried out where methoxzolamide was used as the weak binder and only pifithrin-µ was added. As expected, pifithrin-µ out-competed some of the weak binder that was present, at approximately the same degree as was observed when all 100 compounds in Library C4 were present (Fig. 5b). This experiment confirms that pifithrin-µ was responsible for the positive hit in the assay. Furthermore, because the signal for the reporter ion, when only pifithrin-µ was present, is equal to the signal of the reporter ion of the 100 compound mixture containing pifithrin-µ, no other compound in the 100 compound library could have been a strong binder. (If two strong binders were present in the well, the reporter ion signal for the 100 compound library would be appreciably larger than it is.) Pifithrin-µ itself is not ionizable, so it would not be detectable by ESI-MS in either in the positive or negative mode. This property likely explains why it had not previously been identified as a CA inhibitor, even though MS-based HTS assays, screening over 100,000 compounds had been conducted previously using CA as a target protein^25^.

What if the active compounds’ identity cannot be inferred based on structure? A third approach to the identification of any binding compound – whether it ionizes or not–would be to continue to split and rescreen the active wells. In the worst possible case, the inhibitor could be positively identified in four rounds of screening, by testing a total of 18 wells. For example, the following split-and-rescreen strategy could be used: First, split the 400 compounds into 5 wells of 80 compounds each and re-screen; for the active well, split the 80 compounds into 5 wells of 16 and re-screen; split that active well into 4 wells of 4 compounds and screen; split the active well and screen each of the four compounds individually. In this manner, only 18 additional wells are tested. Clearly, several options are available to identify the active compound in any well, and researchers can choose the appropriate approach based on the individual circumstances of their screen.

Is pifithrin-µ a new CA inhibitor, or is it a false positive hit? To answer this question, an inihibition assay was carried out using a standard protocol where the substrate of carbonic anhydrase, carbon dioxide, is transformed to carbonic acid, and this conversion is monitored by a change in pH of a buffered solution. Pifithrin-µ was tested for its inhibitory ability, and it dramatically impacted the activity of carbonic anhydrase, even at low concentrations. The IC_50_ was determined to be ~25 nM (see Supporting Fig. 2). These results confirm that pifithrin-µ is a newly-identified inhibitor of CA. CA inhibitors are highly sought-after for the development of a variety of drug products, treating diseases as broad as cancer to glaucoma^29–32^. This new lead, therefore, expands the CA structures that can be considered for drug development.

## Conclusion

We developed a new MS-based HTS method that has two key advantages over existing methods: First, the assay evades false positives, since compounds that bind nonspecifically do not out-compete the weak binding ligand at the protein’s binding site; second, since the method does not detect binding ligands directly, but rather detects when a ligand out-competes a weaker binder, even non-ionizing compounds can be profiled; therefore, false negatives are mitigated as well. The assay is rapid, screening 400 compounds in 10 minutes, and it requires only minute quantities (pmols to nmols) of target protein. After demonstrating that the assay had appropriate discriminatory power for three different proteins, CA was screened against a library of 1200 compounds and one known strong binder of the targets. We detected the spiked strong binder each time it was present, and none of the 1200 compounds tested gave a false positive response. Furthermore, a new inhibitor, pifithrin-µ, was identified; this is a surprising and exciting result, considering that CA inhibitors are therapeutically valuable drug targets for a number of diseases. This rapid and simple assay can be implemented on a variety of protein targets; binding ligands with a broad range of binding affinities can be detected.

## Electronic supplementary material

Supporting Information
