# Supplementary material for: Rapid LC-MS Based High-Throughput Screening Method, Affording No False Positives or False Negatives, Identifies a New Inhibitor for Carbonic Anhydrase
Source: Sci Rep. 2017 Sep 4;7:10324. doi: 10.1038/s41598-017-08602-w (PMC5583356; doi:10.1038/s41598-017-08602-w)
Supplement: Supplementary file 1 — Supporting Information [file 41598_2017_8602_MOESM1_ESM.doc]

**Supporting information**

**Rapid LC-MS Based High-Throughput Screening Method, Affording No False Positives or False Negatives, Identifies a New** **Inhibitor for Carbonic Anhydrase**

Kasun P. Imaduwage, Jude Lakbub, Eden P. Go, and Heather Desaire

The Ralph N. Adams Institute for Bioanalytical Chemistry and Department of Chemistry, University of Kansas, 2030 Becker Drive, Lawrence, KS 66047, USA

Corresponding author: hdesaire@ku.edu

**Supporting Figure 1**: Structures of the known CA inhibitors and the unknown CA binder pifithrin-µ. Similar functional groups were circled in red in all the structures, except dash lines were used on pifithrin-µ.

**Supporting Figure 2**: The plot of % inhibition versus the log concentration of the inhibitor. IC50 value (~ 25 nM) for pifithrin-µ compound identified as carbonic anhydrase inhibitor in the LOPAC1280 library.


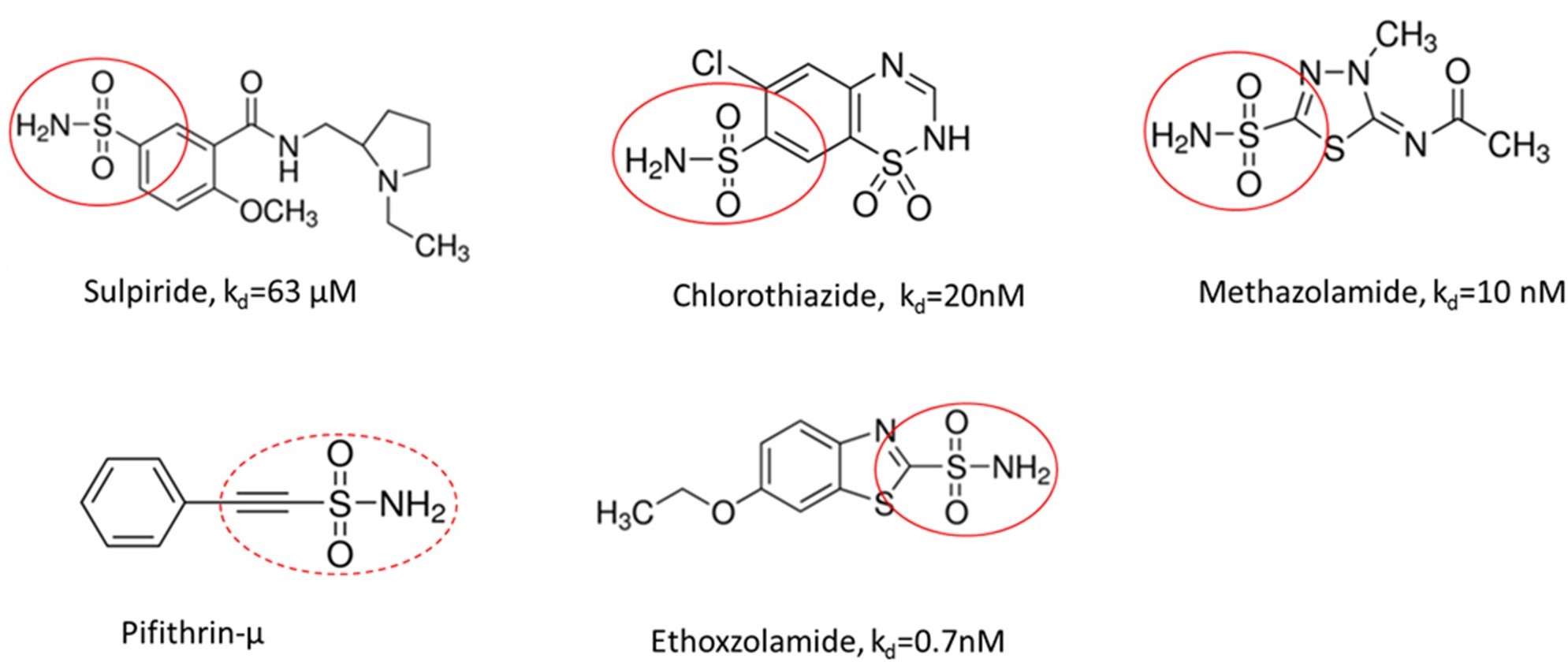


**Supporting Figure1**:


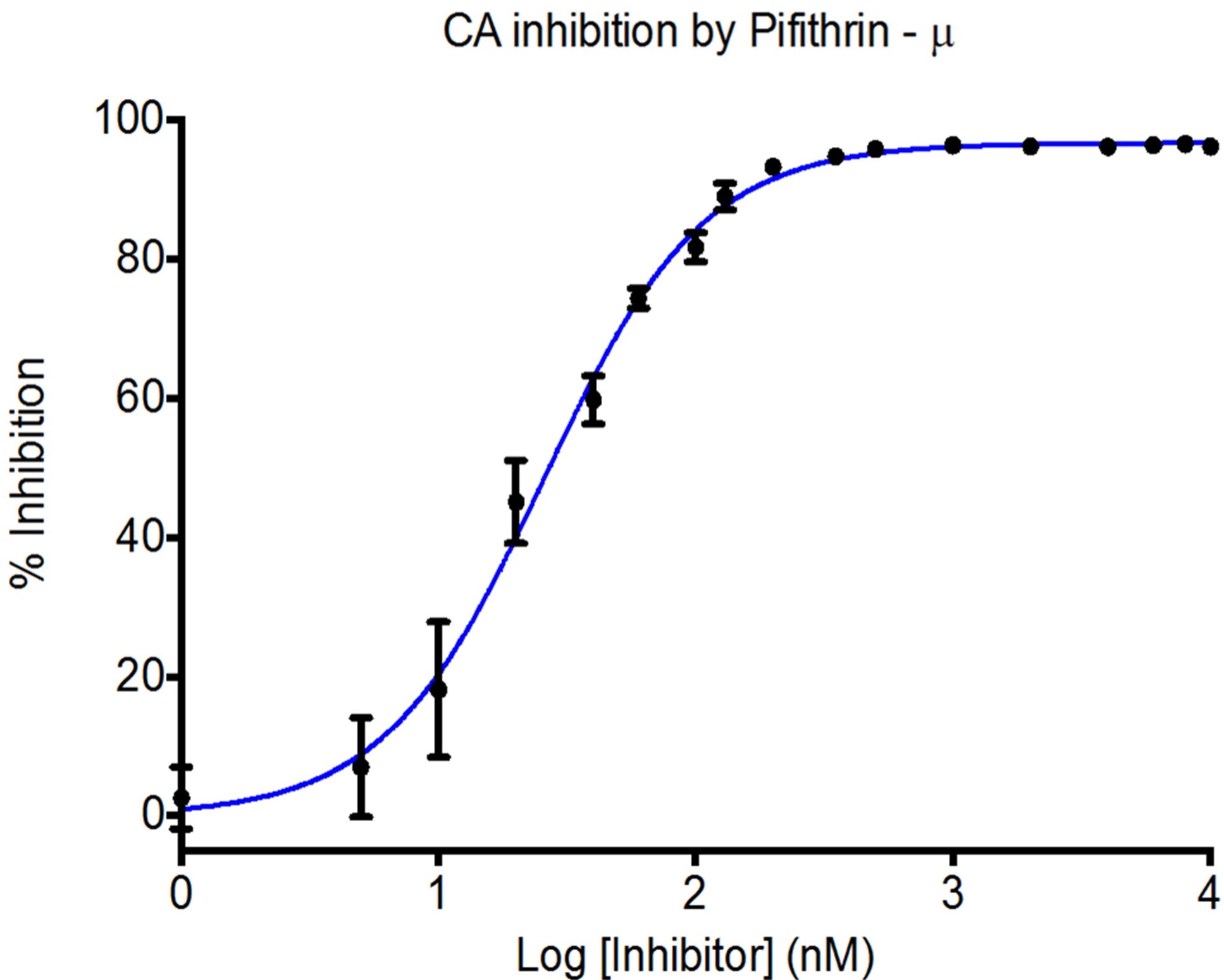


**Supporting Figure 2**:
